# A Plasma Metabolomic Profiling of Exudative Age-Related Macular Degeneration Showing Carnosine and Mitochondrial Deficiencies

**DOI:** 10.3390/jcm9030631

**Published:** 2020-02-27

**Authors:** Juan M. Chao de la Barca, Barnabé Rondet-Courbis, Marc Ferré, Jeanne Muller, Adrien Buisset, Stéphanie Leruez, Guillaume Plubeau, Thibaut Macé, Laurie Moureauzeau, Stéphanie Chupin, Lydie Tessier, Odile Blanchet, Guy Lenaers, Vincent Procaccio, Delphine Mirebeau-Prunier, Gilles Simard, Philippe Gohier, Dan Miléa, Pascal Reynier

**Affiliations:** 1 Département de Biochimie et Génétique, Centre Hospitalier Universitaire, 49933 Angers, France; JMChaoDeLaBarca@chu-angers.fr (J.M.C.d.l.B.); Stephanie.Chupin@chu-angers.fr (S.C.); LyTessier@chu-angers.fr (L.T.); ViProcaccio@chu-angers.fr (V.P.); DePrunier@chu-angers.fr (D.M.-P.); GiSimard@chu-angers.fr (G.S.); 2 Unité Mixte de Recherche MITOVASC, équipe Mitolab, Centre National de la Recherche Scientifique 6015, Institut National de la Santé et de la Recherche Médicale U1083, Université d’Angers, 49933 Angers, France; marc.ferre@univ-angers.fr (M.F.); guy.lenaers@inserm.fr (G.L.); 3 Département d’Ophtalmologie, Centre Hospitalier Universitaire, 49933 Angers, France; ophtalmopontarlier@gmail.com (B.R.-C.); Jeanne.Muller@chu-angers.fr (J.M.); Adrien.Buisset@chu-angers.fr (A.B.); stephanieleruez@hotmail.fr (S.L.); Guillaume.Plubeau@chu-angers.fr (G.P.); Thibault.Mace@chu-angers.fr (T.M.); Laurie.Mourozeau@chu-angers.fr (L.M.); PhGohier@chu-angers.fr (P.G.); dan.milea@snec.com.sg (D.M.); 4 Centre de Ressources Biologiques, BB-0033-00038, Centre Hospitalier Universitaire, 49933 Angers, France; OdBlanchet@chu-angers.fr; 5 Singapore Eye Research Institute, Singapore National Eye Centre, Duke-NUS Medical School, Singapore 168751, Singapore

**Keywords:** age-related macular degeneration, lipidomics, metabolomics

## Abstract

To determine the plasma metabolomic profile of exudative age-related macular degeneration (AMD), we performed a targeted metabolomics study on the plasma from patients (*n* = 40, mean age = 81.1) compared to an age- and sex-matched control group (*n* = 40, mean age = 81.8). All included patients had documented exudative AMD, causing significant visual loss (mean logMAR visual acuity = 0.63), compared to the control group. Patients and controls did not differ in terms of body mass index and co-morbidities. Among the 188 metabolites analyzed, 150 (79.8%) were accurately measured. The concentrations of 18 metabolites were significantly modified in the AMD group, but only six of them remained significantly different after Benjamini–Hochberg correction. Valine, lysine, carnitine, valerylcarnitine and proline were increased, while carnosine, a dipeptide disclosing anti-oxidant and anti-glycating properties, was, on average, reduced by 50% in AMD compared to controls. Moreover, carnosine was undetectable for 49% of AMD patients compared to 18% in the control group (*p*-value = 0.0035). Carnitine is involved in the transfer of fatty acids within the mitochondria; proline, lysine and valerylcarnitine are substrates for mitochondrial electrons transferring flavoproteins, and proline is one of the main metabolites supplying energy to the retina. Overall, our results reveal six new metabolites involved in the plasma metabolomic profile of exudative AMD, suggesting mitochondrial energetic impairments and carnosine deficiency.

## 1. Introduction

Age-related macular degeneration (AMD) is one of the leading causes of visual impairment in the elderly [1], affecting 30–50 million individuals worldwide [2]. AMD results from a complex combination of genetic, lifestyle and environmental factors, such as light damage, smoking and nutritional habits. At an early stage, the disease is characterized by drusen and pigmentary changes, whereas dry (atrophic) and wet (neovascular or exudative) subtypes are found at a later stage. The visual prognosis of exudative AMD has been significantly improved by intravitreal injection of anti-vascular endothelial growth factor (anti-VEGF) agents [3], but the metabolic disturbances contributing to the dysfunction of the retinal pigment epithelium (RPE) and, secondarily, the loss of photoreceptors, remain poorly understood [4,5].

The few metabolic studies previously performed in AMD patients were recently reviewed [6]. A first study was performed using untargeted mass spectrometry in the plasma of neovascular AMD patients (*n* = 26) compared to controls (*n* = 19). This study revealed discriminant features such as di- and tri-peptides, covalently modified amino acids, bile acids and vitamin D-related metabolites [7]. Subsequently, an untargeted metabolomics study performed on the plasma of exudative AMD patients (*n* = 20), compared to healthy controls (*n* = 20), identified 10 discriminating metabolites, including amino acids, palmitoylcarnitine, isomaltose, hydrocortisone and biliverdin [8].

Then, two large cohorts from Coimbra (201 patients and 42 controls) and Boston (113 patients and 40 controls) of patients with various stages of severity were investigated using nuclear magnetic resonance [9]. Differences, probably related to nutritional and lifestyle habits, were found between the two cohorts, and only small changes in metabolite concentrations were statistically discriminant between patients and controls, such as for some amino acids, organic acids, creatine, dimethyl sulfone, as well as for some fatty acids and cholesterol-related molecules.

The same team also used mass spectrometry to compare the plasma of early (*n* = 30), intermediate (*n* = 30) and late (*n* = 30) AMD patients with those of control individuals (*n* = 30) [10]. Eighty-seven discriminant metabolites were identified, most of them belonging to lipid pathways, such as acylcarnitines, triacylglycerols and phosphatidylcholines. Further study of the Coimbra and Boston cohorts by mass spectrometry identified 28 metabolites, differing significantly between the patients and the controls, and 67 metabolites differing with the stage of the disease [11]. These discriminant metabolites were mainly related to nucleoside, amino acid, nitric oxide, energetic and phospholipid metabolism.

Lastly, a study targeting the early stages of the disease was recently reported, using a targeted metabolomic approach measuring 188 metabolites in the serum of 72 patients with early or intermediate AMD compared to 72 control individuals [12]. Four metabolite variations were found to be associated to the disease: increased glutamine, increased phosphatidylcholine diacyl C28:1, reduced glutaminolysis rate (aspartate + alanine + glutamate/glutamine ratio) and reduced glutamine/glutamate ratio.

Here, using a standardized targeted metabolomic approach, we compared the plasma of neovascular AMD to those of healthy controls originating from France and uncovered the involvement of six new metabolites, pointing to carnosine and mitochondrial deficiencies.

## 2. Experimental Section

### 2.1. Study Participants

Participants were included in the study from July 2018 to April 2019 after giving their informed written consent for the research. The study was conducted according to the ethical standards of the Helsinki Declaration and its later amendments, and with the approval of the University of Angers ethical committee (Comité de Protection des Personnes, CPP-OUEST 2), agreement number: CB 2013-04.

Individuals were prospectively recruited during routine consultation in the Department of Ophthalmology of the Angers University Hospital, France. All included patients were diagnosed with exudative-AMD (*n* = 40) by a retina specialist (PG); they had their past ophthalmic history recorded, including the number and type of intravitreal injections in the previous three months. Their clinical assessment included visual acuity (using the decimal Monoyer charts converted into logMAR units for statistical analysis), biomicroscopic and fundus examination, IOP measurement, and macular optical coherence tomography (OCT, Cirrus OCT, C. ZEISS Meditec, Dublin, CA, USA). All included patients displayed clinical features of exudative AMD at the time of inclusion, confirmed by macular OCT. Exclusion criteria were concomitant other ophthalmic diseases, including glaucoma, ocular hypertension, past or active uveitis, any vascular retinal disease, or ametropic error with spheric equivalent > +2 diopters or < −6 diopters. Body Mass Index (BMI), other medical history, systemic medications and AMD treatments were also recorded.

Control subjects (*n* = 40) were sex- and age-matched individuals, recruited during routine clinics for mild refractive abnormalities or cataracts at the same department. Each AMD patient was individually matched to a case control subject of the same sex and age (+/− 2 years), recruited during routine consultation in the same Department of Ophthalmology. Inclusion criteria were visual acuity > 20/50, normal biomicroscopic and fundus examination (except a mild cataract), and the absence of any age-related or other maculopathy. In selected cases, macular OCT was performed to rule out an infraclinical maculopathy. Exclusion criteria were the same as in the exudative-AMD group. Systemic treatments as well as vitamin and nutritional supplementations were recorded. The blood samples were collected in the control group after the ophthalmic examination, following the same fasting conditions as in the patients’ group. Statistical analysis of clinical data was performed using the bilateral Student’s t-test for quantitative variables and the χ^2^ test for discrete variables, with between-groups differences considered significant at *p*-values < 0.05.

### 2.2. Samples Collection

The blood samples were collected in all patients and controls after inclusion, when they were requested to return in the morning, and after at least 10 h of fasting. The patient samples were collected at least three weeks after the last anti-VEGF injection. Blood samples were collected in heparin tubes in the Department of Ophthalmology, rapidly transported in crushed ice to the Hospital Biological Resources Center, and immediately centrifuged for 10 min (3000 *g*, +4 °C) before recovery of the supernatant (plasma), which was conserved at −80 °C in 500 μL aliquots until metabolomics analysis.

### 2.3. Metabolomics Analysis

Targeted quantitative metabolomics analysis was carried out using the Biocrates^®^ Absolute IDQ p180 kit (Biocrates Life Sciences AG, Innsbruck, Austria). This kit uses mass spectrometry (QTRAP 5500, SCIEX, Villebon-sur-Yvette, France) to quantify up to 188 different endogenous molecules distributed as follows: free carnitine (C0), 39 acylcarnitines (C), the sum of hexoses (H1), 21 amino acids, 21 biogenic amines and 105 lipids. Lipids are distributed in the kit in four different classes: 14 lysophosphatidylcholines (lysoPC), 38 diacyl-phosphatidylcholines (PC aa), 38 acyl-alkyl-phosphatidylcholines (PC ae) and 15 sphingomyelins (SM). The full list of individual metabolites is provided in Supplementary Table S1. Flow injection analysis, coupled with tandem mass spectrometry (FIA-MS/MS), was used for the analysis of carnitine, acylcarnitines, lipids and hexoses. Liquid chromatography (LC) was used for separating amino acids and biogenic amines before quantitation with mass spectrometry. All reagents used in this analysis were of LC-MS grade and purchased from VWR (Fontenay-sous-Bois, France) and Merck (Molsheim, France). Sample preparation and analysis was performed following the Kit User Manual using the procedure previously described [13].

### 2.4. Statistical Analysis 

Before statistical analysis, the raw data were examined to exclude metabolites with more than 20% of concentration values below the lower limit of quantitation (LLOQ) or above the upper limit of quantitation (ULOQ). However, metabolites with out-of-range concentrations between 20%–40% were kept for the statistical analysis only if the proportion of out-of-range values was significantly different between the AMD and controls. Indeed, excluding these metabolites would increase the rate of false negatives by omitting important information for group discrimination. Principal component analysis (PCA) was used to detect similar patients that group together in the scatter plot and outliers by the analysis of the Hotelling’T2 statistics. Metabolite concentrations were log-transformed before performing Student’s test. To correct for risk type I inflation due to test multiplicity, Benjamini–Hochberg correction was applied to keep the false discovery rate under 10%. PCA was carried out using SIMCA-P^®^ v14.1 software. Univariate analysis was performed using Excel software. 

## 3. Results

### 3.1. Clinical Features of AMD Patients and Controls

Comparisons between demographic, comorbid medical conditions and treatments of individuals with exudative AMD (*n* = 40) and controls (*n* = 40) are presented in Table 1. The mean age of individuals with AMD did not differ significantly from that of controls, nor did the sex ratio. There was no between-group difference regarding thyroid disease, diabetes, hypertension, dyslipidemia, BMI and systemic medications, except for AMD nutritional supplements (*p*-value = 0.012). No control individuals reported taking vitamins or nutritional supplements, whether from medical prescription or self-medication. Only the use of artificial tears, composed of hyaluronic acid, was reported within this group. Unsurprisingly, compared to the control group, the AMD group had significantly lower mean visual acuity (*p*-value < 0.0001), a higher mean central macular thickness (CMT) at the time of the initial diagnosis (*p*-value < 0.0001) and a lower mean CMT at the time of inclusion after the anti-VEGF injections (*p*-value = 0.024). 

### 3.2. Metabolomic Analysis

**Figure 1:**
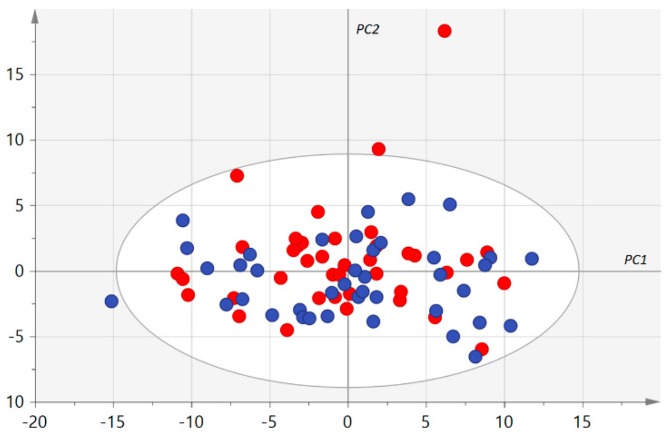
First principal plan of the principal component analysis (PCA). Control and age-related macular degeneration (AMD) samples are represented as blue and red circles, respectively. PC 1, 2: first and second principal components, respectively, are represented using arbitrary units. No group of similar samples can be identified. Only one AMD sample (top right) appears as an outlier but Hotelling’s T2, taking all principal components into account, identified three outliers in the AMD group.

After validation of the kit plate based on QC samples, 38 (20.2%) metabolites were excluded because they were not accurately measured, according to the procedure described in Section 2.4. The statistical analysis was thus carried out on the other 150 (79.8%) metabolites (see Supplementary Table S2 showing the 12,000 measured concentrations). No groups of similar samples plotting together in the first principal plan of the unsupervised PCA were identified (Figure 1). However, three samples from the AMD group were considered as outliers using Hotelling’s T2 statistic. Univariate and supervised multivariate analysis was thus carried out with 77 samples (37 AMD patients and 40 controls). 

**Figure 2:**
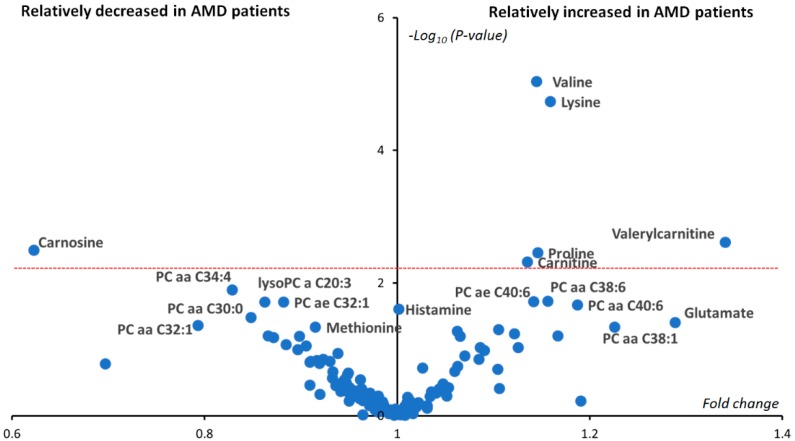
Univariate volcano plot (fold changes versus −log^10^(*p*-values)). Only significant metabolites have been labeled. After applying Benjamini–Hochberg correction, six metabolites (valine, lysine, proline, carnosine, carnitine and valerylcarnitine) situated above the dashed red line were still significantly different between the two groups. Phosphatidylcholines have been labeled as a PC aa (diacyl) or PC ae (alkyl-acyl). The number before the colon indicates the length of the two acyl chains, while the number after the colon indicates the sum of the chains’ unsaturation.

**Figure 3:**
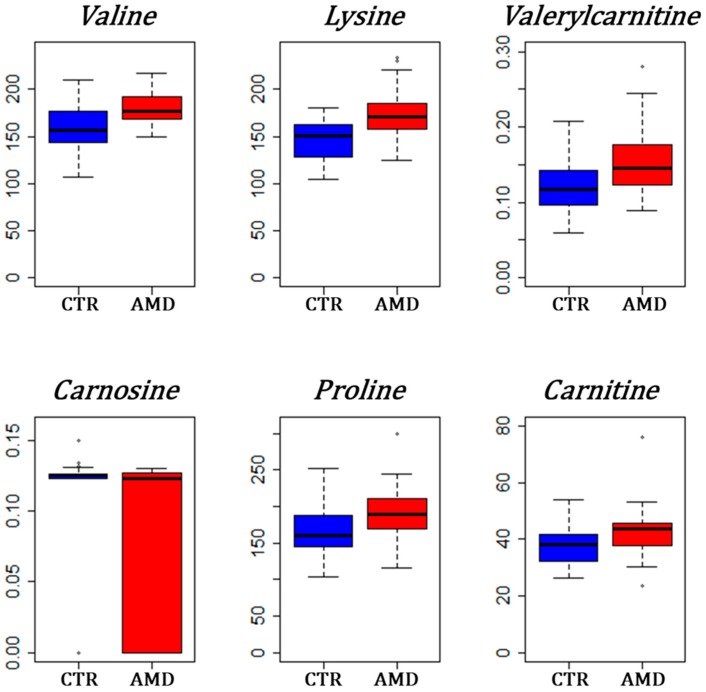
Box plots for the most significant metabolites between AMD and controls (CTR). Groups are represented on the x-axis with the blue box used for controls and the red box for AMD patients. Concentrations are in the y-axis and have dimension μmol/L. The high height of the red box below the median value for carnosine is explained by the high frequency of zero values in the AMD group.

The *p*-values from Student’s *t*-test, comparing 37 AMD and 40 controls, are presented in Figure 2 as a volcano plot combining between-groups fold changes of mean concentration in the x-axis and -*log*_10_(*p*-values) in the y-axis. Only six metabolites remained significantly different between AMD and control groups after Benjamini–Hochberg correction: valine, lysine, valerylcarnitine (C5), carnitine (C0), proline and carnosine. Carnosine was the only metabolite showing reduced concentration in the AMD group with an almost half the mean concentration compared to controls. Carnosine concentration was measured at exactly zero for almost half of AMD patients (18/37 ≈ 49%), whilst only seven controls out of 40 had null measured carnosine concentration (7/40 ≈ 18%, *p*-value = 0.0035). On the other hand, valerylcarnitine (C5) was on average almost 1.4 more concentrated in the plasma of AMD patients compared to controls. Bar plots of the differences between groups of these most important metabolites are given in Figure 3.

## 4. Discussion

Our metabolomic study reveals a significant modification in the concentration of six metabolites in the blood of exudative AMD individuals compared to control individuals. To our knowledge, none of these six metabolites have been reported before in previous AMD metabolomic studies. 

The three amino acids (valine, lysine and proline) with increased plasma concentrations were indirectly related to AMD through a study integrating multiple transcriptomic microarrays data sets [14]. Indeed, among the 15 most significantly enriched gene expression pathways using the Kyoto Encyclopedia of Genes and Genomes (KEGG) algorithm, three involved these amino acids, listed as “valine, leucine and isoleucine degradation”, “arginine and proline metabolism” and “lysine degradation”.

Proline is an important nutrient for RPE [15]. Targeted fluxomics, with _13_C tracers to systematically study nutrient consumption in cultured human fetal RPE [15], showed that proline was consumed faster than any other nutrient, including glucose. In these cells, the Krebs cycle mitochondrial metabolites derived from the proline metabolism were found to be actively transported to the apical retinal side for retina energetic supply. By using _13_C-proline, the same team confirmed in vivo in mice this active flow of energetic proline-derived metabolites toward the retina [16]. They also showed, in an acute mice model of RPE-induced retinal degeneration, that dietary proline was able to protect RPE from oxidative damage, improving the visual function. The changes occurred in the choroid-RPE-retina during ageing, an important mechanism contributing to AMD [17]; the increased concentration of proline in the blood of patients found in our study suggests that a systemic alteration of proline metabolism plays a role in AMD pathophysiology. This hypothesis is reinforced by a recent large-scale Genome Wide Association Study (GWAS), showing that the proline transporter isoform expressed in RPE, SLC6A20 was significantly associated with AMD [18] and by the fact that inborn errors of proline metabolism can result in retinal degeneration.

Carnitine is a dipeptide composed of lysine (also increased in our signature) and methionine, facilitating the transport of fatty acids into the mitochondria to sustain their oxidation. Valerylcarnitine is a short-chain fatty acid (C5) associated with carnitine. The alteration of the carnitine shuttle was already identified in metabolomics studies as a key feature of neovascular AMD [19]. Our study shows that, in addition to the long-chain acylcarnitines already reported, short-chain acylcarnitines such as valerylcarnitine can also be involved, as well as the fatty acids carrier itself, carnitine. 

Electrons can directly enter the mitochondrial respiratory chain, at the level of ubiquinone (Coenzyme Q), through electron transfer flavoproteins that can be fed by fatty acid beta-oxidation as well as by at least nine flavoprotein dehydrogenases present in the mitochondrial matrix. Among these flavoproteins are isovaleryl-CoA dehydrogenase (IVDH), involved in the catabolism of both valerylcarnitine and lysine; glutaryl-CoA dehydrogenase, involved in the catabolism of lysine; and the proline dehydrogenase/oxidase, involved in the catabolism of proline. It is therefore surprising that three of the metabolites that accumulate in AMD are electrons suppliers to the respiratory chain through such electron transfer flavoproteins. Interestingly, reduced levels of riboflavin cofactors are known to induce photoreceptor cell death in mice [20] and alteration of retinal autofluorescence of mitochondrial flavoproteins has been evidenced in patients with AMD [21]. Our results suggest that this mitochondrial flavoproteins dysfunctionality in AMD may be indirectly perceptible in the blood of patients through the accumulation of their substrates. Since these three enzymes use flavin adenine dinucleotide (FAD) as a cofactor, they also suggest a deficiency or an increased consumption of riboflavin (vitamin B2), which is required for FAD synthesis.

Our metabolomic profiling highlights a carnosine deficiency in AMD. Carnosine is a dipeptide composed of beta-alanine and histidine, showing high concentrations in the skeletal muscle and brain. It discloses anti-oxidant and anti-glycating properties [22] and was shown to be protective against aging and neurodegenerative diseases [23]. As increased oxidative stress and advanced glycation end products featuring AMD retina [6], it is tempting to speculate that the relative deficiency in carnosine could contribute to AMD pathogenesis. Since acetylcarnosine has been proposed as treatment in cataract and AMD a decade ago [24], we have carefully verified and excluded the consumption of acetylcarnosine and carnosine by patients and controls.

It is interesting to note that one of the first metabolomic studies in AMD showed the involvement of dipeptides but without their precise identification [7]. Our study identifies carnosine and carnitine as two of these deregulated dipeptides.

Eight phosphatidylcholines and one lysophosphatidylcholine show significant modifications of their concentrations according to the *p*-value but without reaching the *q*-value significance. Modifications of glycerophospholipids have already been reported in the blood of AMD patients [11,12]. Phosphatidylcholines are the main components of circulating lipoproteins that are known to be impaired in AMD [25], thus highlighting their pathophysiological involvement in the disease.

## 5. Conclusions

In conclusion, our study reveals six new metabolites involved in the pathophysiology of exudative AMD. This metabolic profile points to three mechanisms involving the mitochondrial energetic defect: (1) It confirms the alteration in this disease of carnitine shuttle involved in the oxidation and energy supply of fatty acids; (2) it reveals a systemic alteration of proline metabolism which is a crucial metabolite supplying RPE and, secondarily, the retina with energy; (3) it shows that three substrates of mitochondrial electron transfer flavoproteins (lysine, proline and valerylcarnitine) are increased in patients’ blood. This profile, evoking a mitochondrial dysfunction, is fully consistent with previous findings showing multiple mitochondrial dysfunctions in the retinal pigment epithelium of AMD that have been recently reviewed [26]. Our study suggests a systemic origin, perceptible in the plasma, of the mitochondrial impairment. Finally, it reveals a relative deficiency in carnosine, a molecule known to enhance mitochondrial activities in the brain [27] and to protect the retina through its anti-oxidant and anti-glycation properties, thus opening possible therapeutic perspectives.

## Figures and Tables

**Table 1:** Characteristics of participants and ophthalmic management. Population with exudative age-related maculopathy (AMD) compared to controls. BMI: body mass index (kg/m^2^); IOP: intraocular pressure (mmHg); CMT: central macular thickness (µm); VEGF: vascular growth endothelial factor; DHA: docosahexaenoic acid; EPA: eicosapentaenoic acid; DPA: docosapentaenoic acid. *: significant *p*-values < 0.05.

	AMD	Controls	*p*-Values
	N = 40	N = 40	
Demographic data and comorbidities			
Mean age (years)	81.1	81.8	0.62
Females (%)	61	59	0.86
Mean BMI (kg/m^2^)	25.8	25.5	0.76
Thyroid disease (%)	6 (14.6%)	6 (14.6%)	1
Diabetes (%)	1 (2.44%)	0 (0%)	1
Hypertension (%)	24 (58.6%)	22 (53.7%)	0.72
Dyslipidemia (%)	10 (24.4%)	8 (19.5%)	0.35
Systemic medications			
Antihypertensives (%)	23 (56.1%)	21 (51.2%)	0.66
Lipid-lowering medications (%)	12 (29.3%)	8 (19.5%)	0.35
Antiplatelet treatment (%)	7 (17.1%)	9 (22.0%)	0.57
Insulin (%)	0 (0%)	0 (0%)	1
Oral diabetes medications (%)	1 (2.44%)	0 (0%)	1
Corticosteroids (%)	1 (2.44%)	0 (0%)	1
Thyroid hormones (%)	6 (14.6%)	7 (17.1%)	0.76
Estrogens (%)	0 (0%)	0 (0%)	1
Vitamin D (%)	4 (9.76%)	0 (0%)	0.12
Ophthalmological features and AMD management			
Mean visual acuity (logMAR)	0.635	0.136	**<0.0001** *
Mean IOP (mmHg)	14.2	15.5	0.1
Mean CMT (µm) at the initial stage of the disease	332.5	263.6	**<0.0001** *
Mean CMT (µm) at inclusion	236.5	263.6	**0.024** *
Cataract (%)	32.5	27.5	0.808
AMD Management			
-Anti-VEGF intravitreal injections (%)	40 (100%)	—	—
-Nutritional supplementations:			
-Vitamin E	6 (14.6%)	0 (0%)	**0.023** *
-Vitamin C	6 (14.6%)	0 (0%)	**0.023** *
-Vitamin B3, B6, B2, B1, B9, D3, B12	1 (2.44%)	0 (0%)	1
-Zinc	6 (14.6%)	0 (0%)	**0.023** *
-Copper	2 (4.88%)	0 (0%)	0.49
-Selenium	1 (2.44%)	0 (0%)	1
-Manganese	1 (2.44%)	0 (0%)	1
-Omega 3	6 (14.6%)	0 (0%)	**0.023** *
-DHA	6 (14.6%)	0 (0%)	**0.023** *
-EPA	2 (4.88%)	0 (0%)	0.49
-DPA	2 (4.88%)	0 (0%)	0.49
-Resveratrol	1 (2.44%)	0 (0%)	1
-Lutein	6 (14.6%)	0 (0%)	**0.023** *
-Zeaxanthin	6 (14.6%)	0 (0%)	**0.023** *

## Supplementary Materials

The following are available online at https://www.mdpi.com/2077-0383/9/3/631/s1, Table S1: Biochemical families of metabolites quantified in Biocrates^®^ Absolute IDQ p180 kit. Table S2: raw metabolomic data.
