# Supplementary material for: A Plasma Metabolomic Profiling of Exudative Age-Related Macular Degeneration Showing Carnosine and Mitochondrial Deficiencies
Source: J Clin Med. 2020 Feb 27;9(3):631. doi: 10.3390/jcm9030631 (PMC7141125; doi:10.3390/jcm9030631)
Supplement: Supplementary file 1 [file jcm-09-00631-s001.zip › Supplementary Table S1.doc]

**Supplementary Table S1**

**A plasma metabolomic profiling of exudative age-related macular degeneration showing carnosine and mitochondrial deficiencies**

Juan Manuel Chao de la Barca, Barnabé Rondet-Courbis, Marc Ferré, Jeanne Muller, Adrien Buisset, Stéphanie Leruez, Guillaume Plubeau, Thibaut Macé, Laurie Moureauzeau, Stéphanie Chupin, Lydie Tessier, Odile Blanchet, Guy Lenaers, Vincent Procaccio, Delphine Mirebeau-Prunier, Gilles Simard, Philippe Gohier, Dan Miléa, Pascal Reynier

| **Metabolite family** | | | **Number of metabolites** | **Method used for quantitation** |
| --- | --- | --- | --- | --- |
| **Carnitine (C0) and acyl-carnitines (AC)** | | | 40 | FIA-MS/MS |
| **Amino acid (AA)** | | | 21 | LC-MS/MS |
| **Biogenic amine (BA)** | | | 21 | LC-MS/MS |
| **Hexose (H1)** | | | 1 | FIA-MS/MS |
| **Lipids** | **Lysophosphatidylcholine (lyso PC)** | | 14 | FIA-MS/MS |
|  | **Phosphatidylcholine (PC)** | **Diacyl-PC (PC aa)** | 38 | FIA-MS/MS |
|  |  | **Alkyl-acyl-PC (PC ae)** | 38 | FIA-MS/MS |
|  | **Sphingomyelins (SM)** | | 15 | FIA-MS/MS |

**Supplementary Table 1.** Biochemical families of metabolites quantified in Biocrates® Absolute IDQ p180 kit. FIA-MS/MS: Flow Injection Analysis-Tandem mass spectrometry. LC-MS/MS: Liquid Chromatography-Tandem mass spectrometry.
